# Advancing innovation for vaccine manufacturers from developing countries: Prioritization, barriers, opportunities

**DOI:** 10.1016/j.vaccine.2020.12.085

**Published:** 2021-02-22

**Authors:** Benoit Hayman, Alex Bowles, Beth Evans, Elizabeth Eyermann, Lyudmila Nepomnyashchiy, Sonia Pagliusi

**Affiliations:** a DCVMN International, Route de Crassier 7, 1262 Nyon, Switzerland; b Clinton Health Access Initiative, 383 Dorchester Ave, Suite 400, Boston, MA 02127, USA

**Keywords:** Vaccine development, Innovation financing, Developing countries, Partnerships, Regulatory barriers

## Abstract

Development of novel vaccines and improving existing vaccines is critical to addressing areas of unmet or under-addressed health needs globally and to improving existing vaccination coverage and equity. However, vaccine innovation is costly and highly complex. To understand how vaccine manufacturers from developing countries approach innovation, a survey was conducted among company members of the Developing Countries Vaccine Manufacturers Network, in collaboration with the Clinton Health Access Initiative. The survey confirmed that vaccine manufacturers from developing countries are committed to vaccine innovation: 95% of respondents have interest in pursuing vaccine innovation, with strategies targeted towards supplying to low- and middle-income countries. Key barriers to innovation were also surveyed, with respondents highlighting challenges regarding access to in-licensing or joint venture partnerships, financing, and regulatory barriers. Opportunities for innovation are also discussed.

## Highlights

• 95% of respondents manufacturers have a strategy to pursue innovation.
• Innovation targeted at low- and middle- income countries.
• Partnerships with biotechs and universities key in driving innovation.
• Access to partnerships, in-licensing/ joint ventures seen as barrier to novel vaccines.

## 1 Introduction

The Developing Countries Vaccine Manufacturers Network (DCVMN) is a public-health driven alliance representing vaccine manufacturers from developing countries, as defined by the United Nations’ World Economic Situation and Prospect report [1]. Network manufacturers are engaged in research, development, manufacturing and supply of vaccines for local and international use, aiming to protect all people against known and emerging infectious diseases. In 2019, an internal survey reported that 33 manufacturers from this global Network had 181 vaccine projects in the Research and Development (R&D) pipeline, of which 24 were novel vaccines [2]. Over the past decade these manufacturers have demonstrated an increasing commitment to innovation, looking to develop novel vaccines against emerging disease, in addition to improving product presentation of existing vaccines [3]. R&D focus on innovative vaccines is crucial to increasing immunization coverage and reducing mortality and morbidity from infectious diseases.

The Clinton Health Access Initiative (CHAI) is a global health, non-profit organization committed to saving lives and reducing the burden of disease in low-and middle-income countries. CHAI works to strengthen the capabilities of governments and the private sector to create and sustain high-quality health systems, with a team focusing on improving access to vaccines in low- and middle-income countries. CHAI and DCVMN have collaborated to better understand the innovation challenges faced by vaccine manufacturers from developing countries (hereafter referred to as DCVMs).

As part of this effort, a survey was distributed to members of DCVMN to better understand: 1) the extent to which DCVMs prioritize innovation and for which markets; 2) how these manufacturers engage in innovation; and 3) the key barriers to innovation. In addition to reviewing the output of the survey, this report highlights opportunities for advancing innovation.

## 2 Methods

To understand how vaccine manufacturers from developing countries approach innovation, a 17-question survey (Annex 1) was designed by the CHAI vaccines’ team in consultation with the DCVMN Secretariat with the following objectives:

• Quantify the extent to which innovation is a manufacturer priority.
• Understand how manufacturers engage in the innovation pathway (e.g. stages of product development, partnerships, market focus/priority areas).
• Identify the key barriers to innovation for DCVMs

This survey focused on two key types of vaccine innovation: novel vaccines and improved vaccines.

- Novel vaccines: unique vaccines for diseases with no approved vaccines yet (e.g., Chikungunya, Zika, HIV).

- Improved vaccines: vaccines which offer additional value over existing products in terms of antigen attributes, thermostability, and/or presentation (e.g., rotavirus, pneumococcal vaccine).

On 19th November 2019, the link to the online survey was circulated by email to all 42 members of the Network, at that time, soliciting both quantitative and qualitative responses. The survey closed on the 19th February 2020. The data was then analysed to produce the findings presented in this report^1^.

## 3 Results and discussion

The survey had a 48% response rate with twenty company members of DCVMN from 9 different countries responding anonymously to the survey, providing complete or nearly-complete responses.

### 3.1 Innovation

#### 3.1.1 Interest and involvement in innovation

Vaccine manufacturing is complex, and significant time and costs are associated with product development. Advancing a vaccine from pre-clinical development to launching has a very large range estimated risk-adjusted cost of between 18.1 million to 1 billion USD [4] depending on multiple factors. Results from the survey indicate that almost 95% of respondents currently have a strategy to pursue development of completely novel vaccines. This commitment is reflected in respondents’ R&D investment: the arithmetic mean of the reported percentages of overall investments dedicated to novel and improved vaccines was approximately 35%– with a roughly 50–50 split across the two types of innovation (Table. 1). This is particularly interesting given reports showing a decline in multinational corporations (MNCs) innovation output [5].

**Table 1:** Reported means of Investment in percentage) and In-licensing for Vaccine Innovation for the sampled manufacturers.

A) Percentage of overall investments dedicated to novel vaccines	16.63%
B) Percentage of overall investments dedicated to improved vaccines	18.00%
C) Percentage of innovation from in-licensing	20.83%
D) Percentage of in-licensing having evidence of efficacy in humans at the point of licensing	38.91%

DCVMN member manufacturers were asked questions A-D in order to quantitatively identify how much manufacturers invest in investing in novel and improved vaccines, and also the role innovation plays in this investment in innovation. Exact number of respondents to each question is as follows: A) 16 responses B) 13 responses C) 12 responses D) 11 responses

#### 3.1.2 Target markets for vaccine innovation for DCVMs

**Fig. 1:**
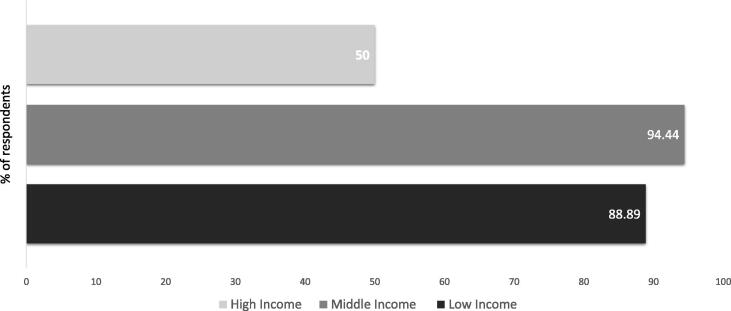
DCVMN member manufacturers were asked in which of the following markets does your organization have a strategy to pursue innovation; low income, middle income, high income. In total 18 manufacturers responded to this question.

DCVMs are motivated by their mission to protect people from known and emerging infectious diseases and to produce innovative vaccines, particularly targeted towards low- and middle-income economies. Survey results show that approximately 89% of respondents have a strategy to pursue innovation targeting low-income markets while over 94% reported having a strategy to target innovation in middle-income markets (Fig. 1).

In 2018, vaccine manufacturers from developing countries produced over 50% of the vaccine doses procured by UNICEF globally [6]. Such efforts are critical in assuring vaccines are supplied to where they are needed most, such as in countries where the incidence of infectious disease is often the highest. For example, 62% of the world’s unprotected children reside in 10 low- to middle-income countries [7] and nations in sub-Saharan Africa, South Asia and Latin America are at risk from five or more major vector-borne diseases [8]. DCVMN members have a track record of innovations in vaccine product attributes that are important in increasing programmatic ease of use, particularly for low- and middle-income countries [2], [3]. One example is a thermostable rotavirus vaccine: the vaccine’s heat stable characteristics help it maintain safety and efficacy even when the cold chain is compromised – an occurrence more likely in low-income countries where infrastructure is poor [9].

The availability of domestic vaccine supply in these countries is vital in the face of potential outbreaks, as demonstrated during the COVID-19 pandemic period and by the need for global and equitable access to vaccines. Furthermore, dedication to innovation can lead to more novel and improved vaccines achieving WHO prequalification, making them eligible for procurement by UN agencies. This provides a means to increase supply globally, increase affordability, apply economies of scale and expand procurement options [10].

#### 3.1.3 Sources of innovation: in-licensing versus in-house development

In-licensing provides a mechanism for manufacturers to rapidly access expertise and technology that would, as a sole entity, be more expensive and require intensive research or additional technical capabilities [11]. Conversely, in-house product development can avoid some licensing costs and potentially secure a higher proportion of the returns of any successful product, although it requires significant scientific and technical expertise [12].

The survey results showed that only 21% of innovation programs from the respondents are derived from in-licensing agreements (Table. 1). This suggests that many DCVM companies innovate in-house. Further research would be required to determine if this is influenced by challenges with securing opportunities for in-licensing. Sampled manufacturers reported that 39% of this in-licensing occurs with candidate vaccines that have evidence of efficacy in humans at the point of in-licensing (Table. 1). This indicates that vaccines are sometimes taken to clinical trials by another developer before licensing agreements with DCVMs are finalized, reducing financial risk, aligned with common practice in the pharmaceutical industry.

**Fig. 2:**
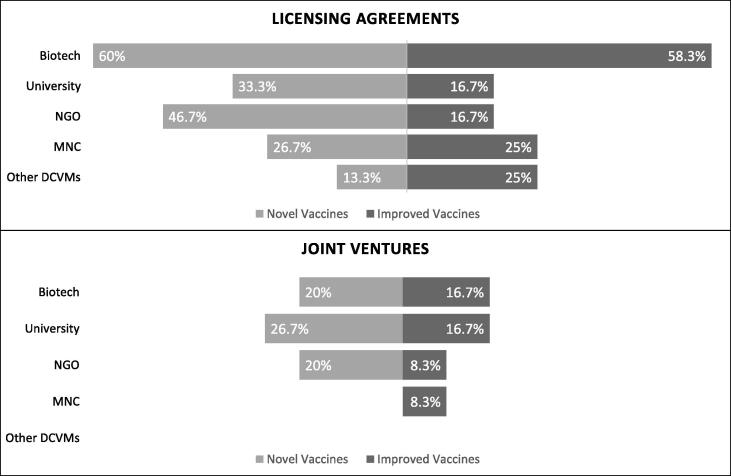
Proportion of emerging manufacturing engaging in licensing agreements and joint ventures.

Licensing agreements and joint ventures are both viable means for manufacturers to access technologies and develop innovative products. Both play a role in reducing the long timeframe and high costs associated with product development [11]. This study assessed both the frequency of such partnerships, and the type of organizations DCVMs engage with. More respondents reported engaging in licensing agreements than engaging in joint ventures, regardless of the type of organization partnered with (biotechnology firms, non-governmental organizations (NGOs), MNCs, other DCVMs) (Fig. 2).

For licensing agreements, biotechnology firms were most common partners for both novel and improved vaccines (with agreements for 60.0% and 58.3% of the sample DCVMs respectively) followed by NGOs (46.7%) and universities (33.3%) for novel vaccines; and MNCs and other DCVMs for improved vaccines (25.0% each).

Joint ventures, when used, were more common for developing novel vaccines than improved vaccines (Fig. 2). Universities were the most frequently partner for joint ventures for novel vaccines (26.7%). For improved vaccines, manufacturers engaging in joint ventures also regularly collaborated with biotechnology firms (16.7% of sample manufacturers). Furthermore, for both novel and improved vaccines there were no joint ventures reported between DCVMs, potentially indicating either an absence of complementary capabilities or the need for a platform for sharing best practices and catalyzing collaborations.

### 3.2 Barriers to investment in novel and improved vaccines

Investing in novel and improved vaccines requires complex decision-making. From high fixed costs and long lead times to biological variability in manufacturing processes and analytical methods, many variables must be taken into consideration [12]. Understanding the key barriers to investment in novel and improved vaccines, as identified by DCVMs themselves, may help international stakeholders to support strategies for innovation. The survey assessed the key barriers to investment in innovative vaccines, and the key findings are discussed below.

#### 3.2.1 Limited access to in-licensing and other partnership opportunities

**Fig. 3:**
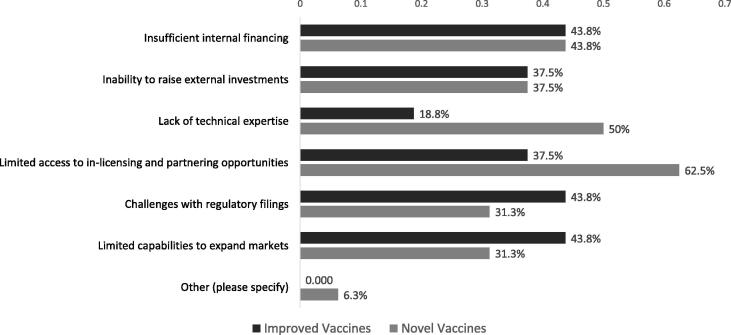
Emerging manufacturers most critical barriers to investment in innovation.

As discussed above, survey results indicate that a majority of innovation by the sampled manufacturers is derived from in-house efforts rather than in-licensing. However, in this survey, 63% of the manufacturers cited limited access to partnerships as a critical barrier to developing novel vaccines, with 38% citing the same barrier for development of improved vaccines (Fig. 3), with the difference likely reflecting the higher technical difficulty of developing novel vaccines [13].

Partnerships, whether with stakeholders, industry or among manufacturers, are instrumental to vaccine development [14], [15]. Often development processes require engagements from multiple collaborators at different stages of development, making formal networks and platforms for sharing best practices and/or candidates or raw materials invaluable [12].

The next most prominent barrier to investment in novel vaccines, reported by 50% of respondents, was a lack of technical expertise, which can include, and is not limited to, scientific, regulatory, and strategic expertise. However, to attain sufficient scientific and technical skills for vaccine manufacturing, low-resource countries are frequently required to import skilled labor, which greatly increases costs [12], [16]. Further research is required to pinpoint the specific technical areas for which support would most benefit DCVMs.

#### 3.2.2 Insufficient financing

Internal and external financing were also highlighted as key challenges for novel vaccine development (by 44% and 38% of respondents, respectively, Fig. 3). A number of international organizations and foundations provide grants, in addition to government subsidies and opportunities to engage in venture capital [14]. However, the results of this survey suggest funding sources may be insufficient or not yet fully optimized by DCVMs. Often innovative products lack a strong commercial case driven by the fact they seldom supply high value markets [17], while the variability of disease epidemiology in target markets also creates demand uncertainties presenting challenges for the development of novel vaccines [13].

#### 3.2.3 Low regulatory capacity

Regulatory filings and limited capabilities to expand markets were recognized slightly more frequently by respondents as important challenges for improved vaccines than novel vaccines (Fig. 3) due to the lack of predictability of timelines for regulatory review and approval of post-approval changes of existing products [18], while new product registrations bring new markets, and thus may be more attractive for manufacturers to engage in. These two factors – expand markets and regulatory filings - likely go hand in hand considering the rigorous regulatory processes that must be completed before a vaccine can be supplied outside the domestic market [12]. Even for WHO prequalified vaccines, the final step of achieving local licensure in a broad range of countries can often be a challenge for DCVMs due to divergent regulatory pathways and country-specific requirements [18], [19]. Regulatory convergence globally would notably remove this barrier [19]. Despite some global efforts (e.g., WHO collaborative registration procedure (CRP)^2^) challenges with CRP adoption in countries remain [18], [20].

## 4 Conclusion

As suggested by the results of this survey, vaccine manufacturers from developing countries are engaged in innovation and have an appetite to expand their role, driven by the need for novel and improved vaccines to increase global immunization coverage against known and emerging infectious diseases. Overall, 95% of the manufacturers sampled in this study reported having a strategy to pursue completely novel vaccines. However, barriers continue to hamper the success of these strategies. For example, despite the benefit of collaborations to advance innovation [14], respondents identified limited access to in-licensing and partnerships as the key barrier to investing in novel vaccines.

Platforms to advance collaboration between academia, international stakeholders and DCVMs will be critical – providing a means for manufacturers to obtain the scientific and technical expertise as well as partners which are beneficial for novel vaccine development^3^^,^ 4. Importantly, the sampled manufacturers have innovative strategies targeted for low- and middle-income markets, where infectious diseases’ burden is the greatest. However, the lack of commercial case often limits investments and stalls the development of needed novel and improved vaccines [13], [16]. Moving forward manufacturers could enhance the capacity to build more detailed commercial insights, such as business cases and demand forecasting and careful cost structuring to incentivize more funding.

This study served to delineate the innovation *appetite* of vaccine manufacturers from developing countries, discussing the priorities, barriers and opportunities surrounding innovation. Future studies are challenged to uncover the causes of the barriers and to provide solutions to overcome these barriers and unlock further innovation, with the goal of increasing access to affordable novel and improved vaccines, particularly for low- and middle-income countries.

## Declaration of Competing Interest

The authors declare that they have no known competing financial interests or personal relationships that could have appeared to influence the work reported in this paper.

## Footnotes

1 Not all vaccine manufacturers from developing countries are members in the Developing Countries Vaccine Manufacturers Network (DCVMN). The findings of this report are strictly based on the sampled manufacturers from DCVMN

2 https://extranet.who.int/prequal/content/collaborative-procedure-accelerated-registration

3 Future Vaccine Manufacturing Research Hub https://www.imperial.ac.uk/future-vaccine-hub

4 Vax-Hub https://www.ucl.ac.uk/biochemical-engineering/research/research-and-training-centres/vax-hub

## References

[1] World Economic Situation and Prospect 2019. United Nations. Available at [Accessed 10 December 2020]. https://www.un.org/development/desa/dpad/wp-content/uploads/sites/45/WESP2019_BOOK-web.pdf.

[2] Hayman B., Pagliusi S. (2020). Emerging vaccine manufacturers are innovating for the next decade. Vaccine X.

[3] Pagliusi S., Dennehy M., Homma A., 2020. Two decades of vaccine innovations for global public good: Report of the Developing Countries’ Vaccine Manufacturers Network 20th meeting, 21-23 october 2019, Rio de Janeiro, Brazil. Vaccine. DOI: 10.1016/j.vaccine.2020.05.062. PMC7286646 32535016

[4] Gouglas D. et al., 2018. Estimating the cost of vaccine development against epidemic infectious diseases: a cost minimisation study. Lancet Glob Health 2018 Dec; 6(12):e1386-e1396. DOI: 10.1016/S2214-109X(18)30346-2 PMC7164811 30342925

[5] Azimi T. (2019). Refueling the innovation engine in vaccines. https://www.mckinsey.com/industries/pharmaceuticals-and-medical-products/our-insights/refueling-the-innovation-engine-in-vaccines.

[6] UNICEF. (2018). Supply Annual Report. Available at [Accessed 22 October 2020]. https://www.unicef.org/media/56226/file/UNICEF%20supply%20annual%20report%202018%20.pdf.

[7] World Health Organization, 2019. Progress and Challenges with Achieving Universal Immunization Coverage. Available at [Accessed 22 October 2020]. https://www.who.int/immunization/monitoring_surveillance/who-immuniz.pdf?ua=1.

[8] Golding (2015). Integrating vector control across diseases. BioMed Central.

[9] Naik (2017). Stability of heat stable, live attenuated Rotavirus vaccine (ROTASIIL®). Vaccine.

[10] Kaddar M., Milstien J., Schmitt S. (2014). Impact of BRICS’ investment in vaccine development on the global vaccine market. Bull. World Health Organisat..

[11] Simonet D. (2002). Licensing agreements in the pharmaceutical industry. J Med Market.

[12] Plotkin S., Robinson J.M., Cunningham G., Iqbal R., Larsen S. (2017). The complexity and cost of vaccine manufacturing – An overview. Vaccine.

[13] Heaton P.M. (2020). Challenges of Developing Novel Vaccines With Particular Global Health Importance. Front Immunol.

[14] Pagliusi S., Che Y., Dong S. (2019). The art of partnerships for vaccines (Conference report). Vaccine.

[15] Tiffay K. (2015). The Evolution of the Meningitis Vaccine Project. Clin Infect Dis.

[16] Luter N. (2017). An updated methodology to review developing-country vaccine manufacturer viability. Vaccine.

[17] Milstien J. (2009). Challenges and potential solutions to innovative vaccine development for developing countries. Procedia Vaccinol.

[18] Dellepiane N. (2020). Alignment in post-approval changes (PAC) guidelines in emerging countries may increase timely access to vaccines: An illustrative assessment by manufacturers. Vaccine X.

[19] Dellepiane N., Pagluisi S. (2018). Challenges for the Registration of Vaccines in Emerging Countries: Differences in Dossier Requirements, Application and Evaluation Processes. Vaccine.

[20] Dellepiane N., Pagliusi S. (2019). Regulatory Experts Working Group Opportunities for improving access to vaccines in emerging countries through efficient and aligned registration procedures: An industry perspective. Vaccine.

