# Supplementary material for: Advancing innovation for vaccine manufacturers from developing countries: Prioritization, barriers, opportunities
Source: Vaccine. 2021 Feb 22;39(8):1190–4. doi: 10.1016/j.vaccine.2020.12.085 (PMC7909323; doi:10.1016/j.vaccine.2020.12.085)
Supplement: Supplementary data 1 [file mmc1.docx]

Annex 1:

The survey that generated the findings of this report included the following 17 questions in English language only, and addressed to vaccine manufacturers’ members of DCVMN, to be answered as “text box”, “YES/NO” or “multiple choice” format:

1. Total number of staff :

2. Please indicate your total annual income defined as: investments/grants and/or commercial revenues (estimate in local currency would suffice):

3. Ownership:

[Private]

[Public (state-owned)]

[Mixed]

4. Select your region:

[East Asia]

[Europe]

[Middle East Africa]

[South America]

[South Asia]

5. Does your company have a strategy to pursue innovation defined as the pursuit of completely novel vaccines?

6. Does your company have a strategy to pursue other types of innovation? (e.g., improvements on existing vaccines and/or new technologies for improved delivery)

7. Does your company have a strategy to prioritize innovations for the following markets? (check all that apply):

[Low income markets]

[Middle income markets]

[High income markets]

8. What % of your overall investments do you use for R&D in novel vaccines?

9. What % of your overall investments do you use for R&D in improved vaccines?

10. What % of your innovation comes from in-licensing?

11. What % of your in-licensing has evidence of efficacy in humans at the point of licensing?

12. What are the most critical barriers to investment in novel vaccines for your company? (select top three):

[Insufficient internal financing]

[Inability to raise external investments]

[Lack of technical expertise]

[Limited access to in-licensing and partnering opportunities]

[Challenges with regulatory filings]

[Limited capabilities to expand markets]

[Other (please specify)]

13. What are the most critical barriers to investment in improved vaccines for your company? (Select the top three):

[Insufficient internal financing]

[Inability to raise external investments]

[Lack of technical expertise]

[Limited access to in-licensing and partnering opportunities]

[Challenges with regulatory filings]

[Limited capabilities to expand markets]

[Other (please specify)]

14. For novel vaccines: Do you have examples of licensing agreements and/or joint ventures with any of the following? Please check all relevant boxes:

[Biotech companies - Licensing (including tech transfer)]

[Biotech companies - Joint venture]

[Universities - Licensing (including tech transfer)]

[Universities - Joint venture]

[Non-profit global health organizations - Licensing (including tech transfer)]

[Non-profit global health organizations - Joint venture]

[Multi-national corporations - Licensing (including tech transfer)]

[Multi-national corporations - Joint-venture]

[Other DCVMs - Licensing (including tech transfer)]

[Other DCVMs - Joint venture]

15. For improved vaccines: Do you have examples of licensing agreements and/or joint ventures with any of the following? Please check all relevant boxes.

[Biotech companies - Licensing (including tech transfer)]

[Biotech companies - Joint venture]

[Universities - Licensing (including tech transfer)]

[Universities - Joint venture]

[Non-profit global health organizations - Licensing (including tech transfer)]

[Non-profit global health organizations - Joint venture]

[Multinational corporations - Licensing (including tech transfer)]

[Multinational corporations - Joint-venture]

[Other DCVMs - Licensing (including tech transfer)]

[Other DCVMs - Joint venture]

16. What type of partnerships do you perceive to be mission critical to your company for innovation?

17. If you could change one thing about the innovation landscape, what would it be?
